# A study of the relationship between adverse childhood experiences, life events, and executive function among college students in China

**DOI:** 10.1186/s41155-018-0107-y

**Published:** 2018-10-19

**Authors:** Shanling Ji, Huiping Wang

**Affiliations:** Ludong University, No. 186, Hongqi Zhonglu, Zhifu district, East Campus of Ludong University, Yantai, Shandong China

**Keywords:** Adverse childhood experiences, Life events, Executive function

## Abstract

**Objective:**

The aim of this study was to investigate the effects of adverse childhood experiences and life events on the inhibitory control ability, cognitive flexibility, and working memory of college students.

**Methods:**

The study involved testing the participants using the Adverse Childhood Experiences (ACEs) Questionnaire, the Adolescent Life Events Scale (Adolescent Self-rating Life Events Checklist, ASLEC), and the program of executive functions designed by E-prime software.

**Results:**

The incidence rate of ACEs was 44.8%. ACEs, life events, and inhibition ability were found to have a significant correlation (*r*1 = 0.50, *r*1 = 0.47, *p* < 0.01). In the switching task, the reaction time of the ACEs group was longer than the reaction time of the non-ACEs group (*t* = − 2.55, *p* < 0.05). Low scorers in the ASLEC exhibited lesser reaction times than their high-scoring counterparts in the tasks related to inhibition, switching, and working memory experiments. The regression analysis results showed that ACEs and life events had a possibility rate of 56% in predicting inhibition ability.

**Conclusions:**

The incidence of ACEs was found to be high, and cognitive flexibility is significantly influenced by ACEs. Life events have a significant impact on inhibition ability, cognitive flexibility, and working memory. ACEs and life events were found to be reliable predictors of inhibition ability.

## Background

Executive functions (EF) refers to a complex set of cognitive abilities that underlie adaptive, goal-directed behaviors and enable individuals to override more automatic or established thoughts and responses (Diamond, 2013). It is an important cognitive ability of individuals and is one of the most important factors that keep one’s mental health (Diamond, 2013; Stella Tsermentseli, 2018; Vitiello and Greenfield, 2017). It is directly related to cognitive actions, such as planning, conceptualizing, abstract thinking, decision-making, implementing feedback, scheduling of events, monitoring, and the capacity to consciously control thoughts. Some researchers argue that the EF consists of three parts: inhibitory, cognitive flexibility, and working memory (Miyake et al., 2000a, b). Inhibitory control mainly refers to the ability of individuals to control own attention, thoughts, and emotions and behavior in a proper state (Beattie et al., 2018); it also refers to one’s capacity to suppress internal or external disturbance (Bell and Cuevas, 2012;Miyake and Friedman, 2012). Working memory (WM) refers to the process of coding information in order to adapt to an ongoing task. It also refers to the process of replacing obsolete information with new and more appropriate information (Kane MJ, Brown LH). Cognitive flexibility refers to the ability to focus on transformation, or to switch between different reactions, rules, and strategies; it is an internal attention control process (Diamond, 2002). In fact, cognitive flexibility is based on both of inhibitory control and working memory (Davidson et al., 2006; Diamond, 2002).

Studies have found that the prefrontal cortex (PFC) is the physiological base of EF, and the connectivity of PFC with multiple regions throughout the brain is very important to EF (Miller and Cohen, 2001). Injuries on the prefrontal cortex are known to cause a series of neurological deficits. For example, in the case of the famous Monterey railroad worker named Gage whose prefrontal cortex was badly injured during working, his cognitive function tests, such as the Wisconsin Classification Test (WCST), were much worse after his brain was getting better, while his other tests, such as intelligence quotient, were normal (Miyake et al., 2000a); with the injured PFC, Gage could not control his temper and impulsive behavior and his work efficiency was not as good as before. From this case, many researchers learnt that EF was associated with some specific areas of the brain’s prefrontal cortex. After many studies, researchers had confirmed that prefrontal cortex of human beings was the main neurophysiological basis of inhibitory control, working memory, planning, conceptualizing, abstract thinking, decision-making, implementing feedback, scheduling of events, monitoring, and other kinds of cognitive function (Miyake et al., 2000a, b; Ardila, 2008; Cohen, 2015).

Miyake et al. (2000a, b) confirmed that inhibitory control ability, cognitive flexibility, and working memory are the main parts of EF and they are independent of each other. Moreover, inhibition control is the core of EF, given that it participates in the process of cognitive flexibility and working memory (Rhoads et al., 2018; Miyake et al., 2000a, b).

Studies have shown that the development time of different parts of EF are different. For example, cognitive flexibility begins after the first fifth year (Rhoads et al., 2018;Seguin and Zelazo, 2004). Cognitive flexibility and working memory can develop rapidly between ages 3 and 7 (Diamond, 2002; Vitiello and Greenfield, 2017). Moreover, cognitive flexibility is known to develop significantly even during adolescence, whereas inhibitory control typically reaches its stability during this period (Dahl, 2004). The EF is typically fully developed by the time when individuals are about 12 years old; however, some certain complex parts of EF, such as decision-making and abstract thinking, develop fully at one’s adulthood. Therefore, childhood and adolescence are important periods for the development of EF. Living environment, nutritional status, familial relations, and school education are known to affect it.

Adverse childhood experiences (ACEs) refer to childhood exposure to a set of correlated and potentially traumatic events before age 18 which include emotional neglect, emotional abuse, physical neglect, physical abuse, and sexual abuse and also include parental substance abuse, parent divorce/separation, the incarceration of a household member, alcoholism, drug abuse, domestic violence, separation or divorce of parents, family members with mental disorders or with suicide or criminal records, premature death of parents, and peer bullying (Felitti et al., 1998). These experiences happened before one’s 18 years old, in the form of negative stress factors, may affect the normal development of executive functions by some certain ways. For example, children with such adverse experiences may have more active hypothalamic-pituitary-adrenal (HPA) axis which is the humoral regulation system and the stress response system, making a variety of stress response to adapt the harmful environment into a new homeostasis (López-Caneda et al., 2012; Lee and Rhee, 2017). The overactive HPA axis of children would damage their growing brain and change the normal developing function of brain by poisoning neurons and promoting neuronal apoptosis (Bernstein et al., 1994; van Dalfsen and Markus, 2018; Lee and Rhee, 2017; McKay and Zakzanis, 2010; Mcdermott et al., 2012; Dube et al., 2003; Anacker et al., 2014).

It has also been shown that traumatic childhood events, such as the abuse and ignorement of physical, emotional, and sexual could affect the EF during early adulthood (De Prince et al., 2009; Wamser-Nanney and Sager, 2018; Tashjian et al., 2016). Individuals who have experienced maltreatment might have behavioral problems, decreased cortical brain volumes, and deficits in decision-making ability and cognitive performance (Cowell et al., 2015; Hanson et al., 2015; Polderman et al., 2006). For example, familial trauma was associated with poorer performance of EF of young workers, such as lower efficiency and worse customer feedbacks (DePrince and Kristin, 2009). Additionally, posttraumatic stress disorder (PTSD) is associated with the subtle impairment of executive functions (attention regulation) (Aupperle et al., 2012; Scher et al., 2001).

Most studies about EF and stress were carried out to investigate the relationship between childhood stress and EF. For individuals, adverse childhood experiences were facts that had happened many years ago, while some daily life stress events, such as being first time far and longtime away from home, economic difficulties, facing an important test or relatives’ and friends’ death, and losing lovers, happen at the moment. These negative life events might have a worse impact on psychological functions of individuals than adverse childhood experiences. Someone in economic difficulty might work with poor efficiency and he would face much worse consequence in the later days (Blair et al., 2011).

However, there are not many studies that have investigated whether daily stress events can affect the well-developed EF of young adults (under 25 years old). This study aims to address this lack; in doing so, it lists and discusses the impact of adverse childhood experiences and daily life events on the executive functions of young adults.

## Methods

### Participants

In total, 700 students from two universities in Jinan and Yantai, both cities in Shandong province, China, participated in this study. The university in Yantai city is an ordinary-level university and the university in Jinan city is a key one. Both of their students come from all over China and they can represent the majority of Chinese college students in this era.

The mean age of participants was 18.75 ± 0.98, including 298 girls and 360 boys. The other variables, such as native place, household income, and parents’ occupation, can be seen in Table 1.

**Table 1:** The demographic variables and ACEs

Demographic variables	Number	One-way ANOVA
Age		
16–under 17	16	*F* = 0.47 *P* = 0.876
17–under 18	271	
18–under 19	274	
19–under 20	76	
20 and above	21	
Gender		
Male	360	*F* = 10.28 *P* = 0.001
Female	298	
Native place		
Rural	305	*F* = 1.65 *P* = 0.177
Urban	353	
Father’s occupation		
Unemployment	130	*F* = 0.42 *P* = 0.739
Farmers or migrant workers	449	
Working class	44	
Leadership	35	
Mother’s occupation		
Unemployment	242	*F* = 0.82 *P* = 0.505
Farmers or migrant workers	370	
Working class	34	
Leadership	12	
Household income per month		
Under ¥1000	28	*F* = 2.42 *P* = 0.018
¥1000–under ¥2000	65	
¥2000–under ¥3000	119	
¥3000–under ¥4000	117	
¥4000–under ¥5000	122	
¥5000–under ¥6000	112	
Above ¥6000	95	

Information was gathered from the students through questionnaires by cluster sampling, and the collection rate was 100%. Invalid questionnaires were rejected, and 658 valid questionnaires were examined; the effective rate was 94%.

### Measures

#### Adverse childhood experiences (ACEs)

The ACEs of the participants were assessed using two validated measures. First, the Childhood Trauma Questionnaire (CTQ; Bernstein et al., 1997) was used, which includes five subscales: physical neglect, emotional neglect, physical abuse, emotional abuse, sexual abuse before they were 18, and has good convergent and discriminant validity (Bernstein et al., 1997). The following scores represent the condition under which the traumatic experience of a participant was given a score of 1 (each component had to be within the range specified below): emotional neglect ≥ 15 points, or physical neglect ≥ 10 points, or sexual abuse ≥ 8 points, or physical abuse ≥ 10 points, or emotional abuse ≥ 13 points.

The second part, drawing from the Behavioral Risk Factor Surveillance System (BRFSS) (2009) and the ACEs study, focused on family dysfunction. Felitti’s research is seminal in this context, in that it was the first study to explore this issue (Felitti et al., 1998). The ACEs study focused on parents or family members suffering from chronic alcoholism, mental health, or mental illness; families in which a suicide had occurred; wrongdoer’s families; and families of children and adolescents who had experienced physical or verbal abuse. Each “yes” was given a score of 1.

This study focused specifically on participants who experienced at least one of these ACEs.

#### Adolescent Self-rating Life Events Checklist (ASLEC)

Since the concept of “stress” was introduced by H. Selye, the role of life events as psychosocial stressors has received widespread attention. In 1967, Holme and Rahe compiled the first Social Readjustment Rating Scale (SRRS), which introduced a method to quantify life events (Holme & Rahe, 1967). Because of different nationalities, cultural background, age, gender, and professional group in life, the frequency of the incident, and cognitive were different in the evaluation mode. In this study, the Adolescent Life Events Scale (Adolescent Self-rating Life Events Checklist, ASLEC) was on the basis of sum up literature at home and abroad, combined with the physiological and psychological characteristics of adolescents and family social role, which was compiled in 1987. The questionnaire was composed of 27 negative life events to elicit the psychological reactions of adolescents. They were rated between 1 and 6; the higher a participant scored, the more negative his or her experience of life is.

### Experiment paradigm

The e-prime 2.0 software (Psychological Software Tools, Pittsburgh, PA) was programmed to perform inhibitory control, cognitive flexibility, and working memory and to record the response times of participants and the accuracy of their response. It was presented using a 14-in. Lenovo desktop computer at three behavioral laboratories. The three parts of the experiment were conducted in sequence based on the order of the Latin square—i.e., the experimental procedures were presented according to the ABC, BCA, and CAB sequences.

### Inhibitory control

Go No-Go task that was used in the present study was partly adapted from a task developed by Garavan et al. (1999). The experimental materials consisted of a positive triangle and an inverted triangle. The positive triangle stood for the Go signal, and the inverted triangle stood for the Nogo signal. A total of 20 trials were conducted for the practice experiment, and 60 trials were conducted for the formal experiment (the ratio of Go to Nogo was 42:18). The sequence of the experimental procedure is detailed below. First, a fixation point was presented for 500 ms, then a stimulus was presented for 1000 ms, for which a button response was required from the participant. Finally, an empty screen was presented for 500 ms. In the exercise experiment, the Go signal could be activated using the F key and the Nogo signal with the J key. In the formal experiment, the Go signal appears when the F key is pressed, and the Nogo is not associated with any key.

### Cognitive flexibility

Digital-alphabetical switching task that was used in the present study was partly adapted from a task developed by Taylor et al. (1987). The experimental materials consisted of two kinds of shapes (a star-shaped figure and a circle) and two colors (red and blue). The first stage involved judging the color of the images (red or blue), and the second stage involved judging the shape of the images (star or circle). The third stage involved judging either the color or the shape of the image depending on the cue word given. The first two stages were based on the following procedures: first, a fixation point was presented for 500 ms; then, a stimulus was presented for 1000 ms, followed by a button response. An empty screen was presented for 500 ms. The third stage was based on the following procedure. First, a fixation point and a cue word (shape or color) were presented for 500 ms, followed by a stimulus and a button response for 1000 ms. An empty screen was presented for 500 ms. Sixty trials were conducted at each stage.

### Working memory

N-back that was used in the present study was partly adapted from a task developed by Wechsler (1991). This experiment had 0-back and 1-back levels. Each involved a practice experiment and a formal experiment. There were 10 trials in the practice experiment and 40 trials in each of the two parts on the n-back paradigm. The 0-back experiment was based on the following procedure: first, a fixation point was introduced for a duration of 500 ms, followed by five stimuli; each stimulus was presented for 500 ms. When the sixth stimulation, also presented for 500 ms, was introduced, participants were asked to judge whether the sixth stimulus was the same as the fifth. A button response was used to register their judgment for 1000 ms. An empty screen was presented for 500 ms. The 1-back experiment was conducted in the same as the 0-back experiment; however, the 1-back experiment required participants to judge whether the last stimulus was the same as the fourth stimulus.

### Procedure

The ACEs and ASLEC were used to screen participants. From January to February 2017, and 700 students from two universities filled the two questionnaires. The sampling method was cluster random sampling in class units.

From February to May 2017, we randomly selected 30 subjects with adverse childhood experiences and 30 subjects without adverse childhood experiences or life events from 658 participants. All of the 60 participants participated in the three experiment.

From June to July 2017, we randomly selected 30 subjects with life events and 30 subjects without life events or adverse childhood experiences from the rest of participants who participated in this study. All of the 60 participants participated in the three experiment.

### Statistical analyses

Spss20 was used for data entry; data were deleted if the accuracy rate was lower than 90%. Descriptive statistics, independent sample *T* test, correlation analysis, one-way analysis of variance (ANOVA), and regression analysis were performed on the data.

## Results

### The incidence of ACEs

Analyses were conducted by using spss20. The demographic variables of these 658 participants is showed in the Table 1. At the same time, we performed the one-way analysis of variance (ANOVA) on the demographic variables and ACEs to find out that if participants with different demographic variables have different incidence of ACEs. The result showed that people with different household income per month had different incidence of ACEs (*F* = 2.432, *P* = 0.018).

The descriptive statistics of 658 questionnaires about ACEs are presented in Table 2. The incidence of ACEs is 44.8%. Among them, the number of people who experienced one item accounted for 20.1%, and participants who experienced two items accounted for 8.5%. Participants who experienced three items accounted for 7.8%. Additionally, 55.2% of the participants did not report adverse childhood experiences.

**Table 2:** The incidence of ACEs

ACEs	0	1	2	3	4	5	6	7 and more	Total
Number of people	363	132	56	51	21	14	5	16	658
Percentage	55.2%	20.1%	8.5%	7.8%	3.2%	2.1%	0.8%	2.4%	100%

### The demographic variables and EF

We performed the one-way ANOVA on the demographic variables and EF to find out that if participants with different demographic variables have different EF reaction time and correct rate. The statistics is presented in Table 3. The result showed that people with a different age had different reaction time of 0-back, and males and females had a different reaction time and correct rate of switching task. People with a different mother’s occupation or household income per month had a different correct rate of Go/Nogo.

**Table 3:** The one-way ANOVA of demographic variables and EF

	Gender	Age	Native place	Father’s occupation	Mother’s occupation	Household income per month
Reaction time						
0-back	0.10	4.07*	1.26	2.14	1.06	1.07
1-back	0.05	1.34	0.46	0.44	1.55	0.59
Switching	7.33**	0.99	0.31	0.55	0.60	0.71
Go/Nogo	3.05	1.35	0.04	0.174	0.63	1.13
Correct rate						
0-back	0.05	0.29	2.40	1.14	0.46	0.76
1-back	3.82	2.64	1.48	1.48	0.59	1.90
Switching	16.03***	0.37	.045	0.89	0.18	0.58
Go/Nogo	0.18	0.76	3.75	0.38	8.14***	5.14***

*p < 0.05; **p < 0.01; ***p < 0.001

### The correlation analysis between ACEs, life events, and EF

In this study, the ACEs group consisted of 20 people after the screening (i.e., ACEs score 1 or higher); the non-ACEs group (ACEs score < 1) consisted of 22 people.

The high-life-events group (ASLEC score was greater than M + SD) consisted of 18 people, whereas the low-life-events group (ASLEC score < M-SD) was comprised of 24 people.

Correlation analysis was carried out on the average reaction time and correction rate of the three subsystems of EF, ACEs, and life events; the results are reported in Table 4. There is a significant positive correlation between ACEs and life events in response to inhibitory ability (*r*1 = 0.50, *r*1 = 0.469, *p* < 0.01). This implies that a high ACEs score, or a high incidence of life events, would extend the duration of the inhibitory experiment.

**Table 4:** The correlation analysis between ACEs, life events, and EF

	1	2	3	4	5	6	7	7	9	10
1 ACE	1									
2 Life event	0.17	1								
3 0-back	0.15	0.16	1							
4 1-back	0.10	0.13	0.45**	1						
5 Switching	0.30	0.28	0.33*	0.44**	1					
6 Go/Nogo	0.50**	0.47**	0.11	0.30	0.39*	1				
7 0-back2	−0.19	−0.14	−0.41**	−0.02	−0.12	−0.03	1			
8 1-back2	−0.01	−0.17	− 0.26	−0.13	− 0.03	0.02	0.21	1		
9 Witching2	−0.25	−0.09	−0.22	− 0.21	− 0.52**	− 0.23	0.10	0.28	1	
10 Go/Nogo2	0.01	−0.09	0.29	0.25	0.17	0.07	−0.23	0.00	−0.10	1
M(SD)	1.00 (1.73)	71.10 (22.92)	365.77 (119.69)	388.67 (86.07)	498.52 (70.72)	474.89 (78.84)	0.97 (0.035)	0.95 (0.04)	0.91 (0.06)	0.97 (0.04)

*p < 0.05; **p < 0.01

0-back and 1-back measured task working memory; Switching task measured cognitive flexibility; Go/Nogo measured inhibitory ability. 0-back means the reaction time of 0-back; 1-back means the reaction time of 1-back; witching means the reaction time of witching; Go/Nogo means the reaction time of Go/Nogo. 0-back2 means the correct rate of 0-back; 1-back2 means the correct rate of 1-back; Witching2 means the correct rate of witching; Go/Nogo2 means the correct rate of Go/Nogo

### The effect of ACEs and life events on EF

The independent sample *T* test was conducted for the ACEs group and the non-ACEs group, the high-life-events group and the low-life-events group, and the results are shown in Table 5. The differences between the ACEs group and the non-ACEs group in the switching task reach a significant level, *t* = − 2.55, *p* < 0.05. Compared to the non-ACEs group, the response time of the ACEs group was longer. There was no significant difference between the ACEs group and the non-ACEs group in terms of inhibiting ability and working memory.

**Table 5:** Independent sample *T* test

	Go/Nogo	Switching task	0-back	1-back
ACEs				
ACEs (20)	497.84 (130.43)	526.14 (71.79)	399.98 (132.51)	399.96 (90.522)
Non-ACEs (22)	454.02 (59.20)	473.41 (60.95)	334.68 (99.80)	378.40 (82.58)
*t*	−1.38	−2.55*	−1.79	−0.80
Life events				
High ASLEC (18)	526.34 (122.56)	527.78 (66.80)	399.71 (98.06)	425.47 (93.95)
Low ASLEC (24)	436.30 (58.306)	476.57 (66.67)	340.33 (129.83)	361.06 (69.49)
*t*	3.16**	2.46*	1.69	2.45*

*p < 0.05; **p < 0.01

As is shown in Table 5, there is a significant difference between high-life-events group and low-life-events group in inhibiting ability, *t* = 3.16, *p* < 0.001. The two groups have a significant difference in switching task, *t* = 2.46, *p* < 0.05. There is a significant difference between the two groups in working memory (1-back), *t* = 2.45, *p* < 0.05. In the three experimental tasks, the response time of the high-life-events group is longer than that of the low-life-events group.

### The regression analysis of ACEs and life events on inhibitory ability

As shown in Table 3, ACEs and life events only have a significant correlation with the inhibitory ability of executive functions. Given this, the study attempted to conduct a regression analysis. First, the scores of the life events and the ACEs were decentralized. The average reaction of the inhibiting ability was seen as the dependent variable *Y*, with ACEs and life events as the independent variables *X*_1_, *X*_2_. The demographic variables of participants (age, gender, native place, household income, parents’ occupation, etc.) were translated into dummy variables and then introduced in regression equations. The decentralized ACEs and life events were then separately introduced into the regression equation, thus obtaining the regression equation 1 and regression equation 2 respectively. Finally, the decentralized ACEs and life events were introduced in the regression equation together to obtain regression equation 3. The results are shown in Table 6.

**Table 6:** Regression analysis

Regression	Dependent variables	Independent variables	*β*	*t*	*R* ^2^
Model 1	Inhibiting	ACEs	0.48	2.69*	0.46
Model 2	Inhibiting	life events	0.40	2.74**	0.46
Model 3	Inhibiting	ACEs	0.42	2.48*	0.56
		life events	0.35	2.53*	

*p < 0.05; **p < 0.01

In regression model 1, the regression coefficient of ACEs on inhibition ability was significant (*β* = 0.48,*t* = 2.69, *p* < 0.05), and the *R*^2^ was 0.46. In regression model 2, the regression coefficient of the life event on inhibition was significant (*β* = 0.40, *t* = 2.74, *p* < 0.01), and the *R*^2^ was 0.46. In regression model 3, the regression of ACEs and life events on inhibition ability was significant (*β*_1_ = 0.42, *β*_2_ = 0.35, *t*_1_ = 2.48, *t*_2_ = 2.53), and the *R*^2^ was 0.46.

## Discussion

### The incidence of ACEs

The incidence of ACEs in this study was 44.8%; moreover, 55.2% of the participants did not report the incidence of adverse experiences during childhood. Additionally, the number of participants who reported experiencing one item accounted for 20.1%, and those who reported the experience of two items accounted for 8.5% (Table 2). Felitti founded the incidence of ACEs in his study was 65% (Felitti et al., 1998). Bellis founded in his study more than 50% people had ACEs in European countries and more than 65% people had ACEs in Romania (Bellis, 2014). The different incidence of ACEs among different countries may be related to the cultural characteristics of each country. For example, the divorce of parent is a terrible thing for Chinese children; they will feel sad for losing one of their parents because Chinese live in a collectivist culture and they like to unite all people and strive to make a collective harmonious atmosphere. In this condition, not only did the children of divorced parents experience losing a happy family, they may also experience potential peer insults or bullying for their broken families, making them not only feel sad but also ashamed and guilty. In contrast, in some western countries with high divorce rates, the children whose parents divorced might not have a such terrible feeling for losing one of their parents because they live in a culture of individualism and they think everyone should be responsible for their own affairs and the affairs of others are not their responsibility. Therefore, the incidence rates vary from country to country.

Overall, the incidence of ACEs (i.e., neglect, maltreatment, and family dysfunction) in this study was high. Moreover, participants with a different household income per month had different incidence of ACEs (Table 1). Family economy is an important factor in determining family harmony. Children who grow up in low-income families tend to witness more family quarrels, violence, etc. caused by family financial difficulties. This also increases the likelihood of their ACEs experience (Felitti et al., 1998).

### The relationship between the ACEs, life events, and EF

In Table 3, the result shows that people with a different age had a different reaction time of 0-back; males and females had a different reaction time and correct rate of switching task. People with a different mother’s occupation or household income per month had a different correct rate of Go/Nogo. This implies that the performance of participants could be affected by their demographic variables (i.e., native place, household income, parents’ occupation). In some way, this may happen during their growth experience (Anacker et al., 2014;DePrince and Kristin, 2009; DePrince et al., 2009).

Table 4 shows that there was a significant positive correlation between the ACEs, life events, and inhibitory ability. This implies that participants with high ACEs scores or high-life-events scores would require more time to formulate the right response in inhibition tasks. ACEs and life events, or long-term stress events and recent stress events, are closely related to inhibitory control ability of individuals (Dahl, 2004;Kane et al., 2007; Karbach and Kray, 2009;Taylor et al., 1987).

In addition, working memory and inhibitory ability were significantly positively correlated with switching tasks. It also shows that cognitive flexibility is an important component of EF (Diamond, 2002). In the switching experiment, using the same stimulus materials for different tasks depends not only on the inhibition of other unrelated tasks or interferences, but also on the timely response to task type requests. Therefore, inhibition control and working memory are necessary for cognitive flexibility (Rhoads et al., 2018;Diamond, 2002;Miyake and Friedman, 2012). Some scholars also believe that cognitive flexibility enables actions to change to better suit the contents of working memory as cognitive flexibility involves both inhibitory control and working memory (Diamond,^2002^; Kane et al., 2007; Karbach and Kray, 2009; Miyake and Friedman, 2012; Vitiello and Greenfield, 2017).

### The effect of ACEs and life events on EF

As is shown in Table 5, the differences between the ACEs group and the non-ACEs group occur only in the switching task. The ACEs group’s need is characterized by longer reaction times than the non-ACEs group. That means people with ACEs need more time to make a right reaction in the switching task and have worse cognitive flexibility. ACEs damaged the cognitive flexibility of individuals. Loman MM, Johnson AE, and Westerlund had the same result in their study (Loman et al., 2013).

The high-life-events group and the low-life-events group showed significant differences in the three tasks. The high-life-events group had longer reaction time than the low-life-events group. In other words, the performance of the high-life-events group was worse than that of the low-life-events group. This suggests that life events have a negative effect on the three components of EF. Rahdar and Galvan also found that daily stress had neurobiological effects on the cognitive of adolescents (Rahdar and Galvan, 2014).

This means that the recent stress events (life events) are more likely to have a negative impact on performance of college students than long-term stress events (childhood experiences). This is more likely if recent stress events are negative stress events.

This implicated that the influence of ACEs on executive functions of individuals are affected by other factors, such as psychological development, mental flexibility, social support, independent adjustment, and changes in the environment. Personal growth can mitigate the impact of childhood trauma to self-inflicted (Karbach and Kray, 2009). The life events might be alleviated by self-adjustment or the help from others. Therefore, the results of this study show that life events are more likely to have a negative impact on executive performance than adversity childhood experiences.

### The regression analysis of ACEs and life events on inhibitory ability

According to regression analysis (Table 6), the first regression model was significant, with a *R*^2^ of 0.46. Within this model, ACEs was a significant predictor of inhibiting ability. This means childhood experience of individuals can predict the inhibiting ability in the after years (Carr et al., 2013).

The second regression model was also significant, with a *R*^2^ of 0.46, too. Within this second regression model, life events were also a significant predictor. It is sufficient to say that life events also can predict the inhibiting ability in the after years, as childhood experience does. But the life events is the stress that occurs in the moment while the ACEs happened in a few years ago. This means that both of past and present stress have a 46% account for the inhibiting ability performance. The third regression model was also significant, with a *R*^2^ of 0.56. Within this third model, both of ACEs and life events were significant predictors (β = 0.416, t = 2.497, *p* < 0.05; β = 0.348, t = 2.531, *p* < 0.05).

It is clear to see that the life events as a predictor for inhibitory ability, the same as that of ACEs, should not be ignored in our life. Life events can be some daily life events (i.e., economic difficulties, parents quarrel, relationship problems) or can be some serious emergencies or critical incidents (i.e., an end to a relationship, death of relatives and friends, failure in an important examination).

### Limitations

In this study, we found adverse childhood experiences, as well as the life events, can damage executive functions of individuals. But the sample in this study included only college students. The results cannot be generalized to all groups, such as the workers at the same age of college students. They may not have enough ability to cope with school staff and have to leave to work or muddle through the streets.

In the future, researchers need to consider and study individuals who are not going to school at the age they should be and what caused them to leave school exactly. The reason may be something about uneasy family atmosphere, or poor academic performance, or being expelled from the school for violating the school rules due to lack of self-control.

## Conclusions

The incidence of ACEs among college students is high. There is a significant positive correlation between ACEs and inhibitory ability and life events. This implies that the higher score in the ACEs, or the higher score in life events, the longer it takes for inhibitory responses. In addition, working memory and inhibitory ability are significantly positively correlated with switching tasks. In other words, cognitive flexibility is interrelated with working memory and inhibitory ability in some way, and they are not completely independent.

In this study, it is found that ACEs (as long-term stress events) exert influence only on cognitive flexibility. At the same time, life events (as recent stress events) have a significant influence on inhibition ability, cognitive flexibility, and working memory. While we are concerned about the impact of childhood experiences on executive functions, we should also note that current life events have a more serious impact on individual executive functions. Current life events of individuals can affect their inhibition control, cognitive flexibility, and working memory. This is worth our attention because the current life events will affect our academic performance, work efficiency, and life satisfaction.

Finally, both of ACEs and life events are significant predictors for inhibitory ability. Moreover, life events as a predictor for inhibitory ability, the same as that of ACEs, should not be ignored by all of us in order to improve our inhibition control ability, cognitive flexibility, and working memory and in order to improve our academic performance, work efficiency, life satisfaction.

## Abbreviations

ACEs: Adverse Childhood Experiences
ASLEC: Adolescent Self-rating Life Events Checklist
EF: Executive functions
